# Mass-Like Ground-Glass Opacities in Sarcoidosis: A Rare Presentation Not Previously Described

**DOI:** 10.1155/2018/5686915

**Published:** 2018-08-14

**Authors:** Marie Tominna, Sayf Al-Katib

**Affiliations:** Beaumont Hospital, Oakland University William Beaumont School of Medicine, Department of Diagnostic Radiology and Molecular Imaging, 3601 W 13 Mile Rd, Royal Oak, MI 48073, USA

## Abstract

Various typical and atypical imaging findings for pulmonary sarcoidosis have been described in the literature. Ground-glass opacities are one of the atypical manifestations, reported as diffuse or patchy ill-defined opacities frequently associated with additional findings and interstitial nodules. We performed a literature review to determine if our case had previously been described. The literature describes cases of mass-like consolidations, but there are no reports of mass-like ground-glass opacities. The appearance of the ground-glass opacities in our case is unique, appearing as discrete well-defined mass-like ground-glass opacities in a peribronchovascular distribution without additional parenchymal findings typically seen in sarcoidosis.

## 1. Introduction

Sarcoidosis can present with a myriad of imaging findings in the chest. Typical and atypical manifestations have been described within the literature. Finding ground-glass opacities within the lungs on imaging is one of the atypical imaging features. These ground-glass opacities are typically diffuse or patchy and ill-defined, and frequently associated with additional imaging findings such as interstitial nodules [1, 2]. Ground-glass attenuation correlates with granulomatous lesions in alveolar septa and around small vessels on histopathology, rather than an alveolitis picture [3]. For the first time, we will report a case of discrete and well-defined mass-like ground-glass opacities as a presentation of sarcoidosis.

## 2. Case Presentation

**Figure 1:**
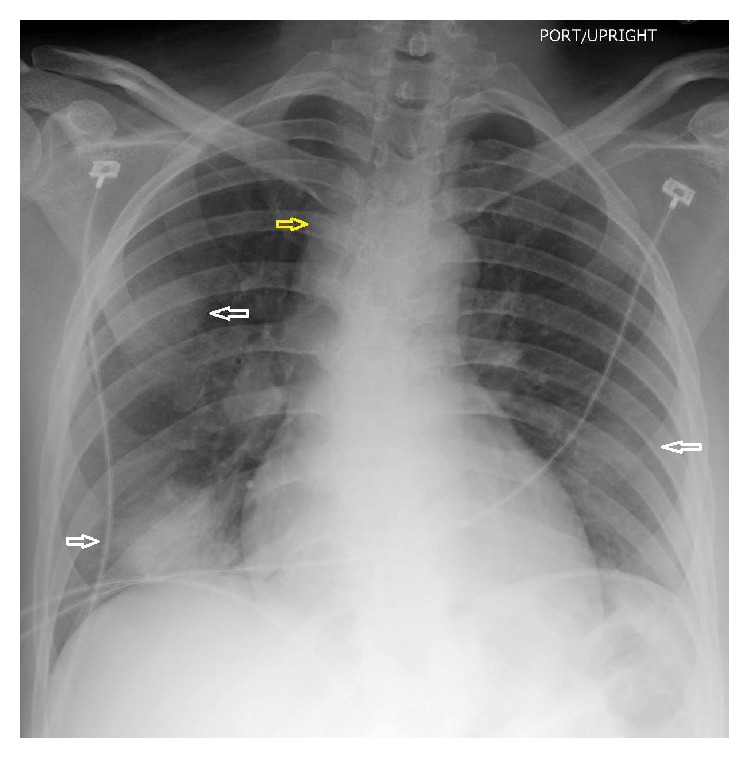
Chest X-ray: multiple bilateral pulmonary masses (white arrows) with right paratracheal lymphadenopathy (yellow arrow).

**Figure 2:**
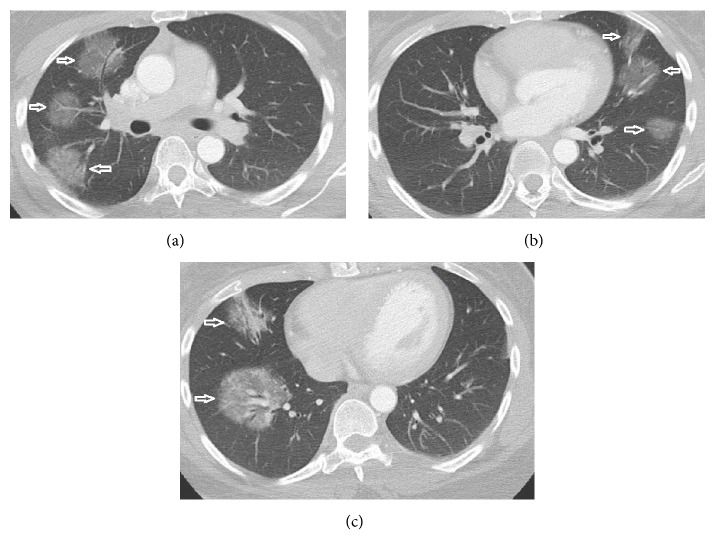
Axial contrast-enhanced CT obtained at different levels (a, b, c), demonstrating bilateral mass-like ground-glass opacities in a peribronchovascular distribution (arrows) with air-bronchograms and vessels seen coursing through.

**Figure 3:**
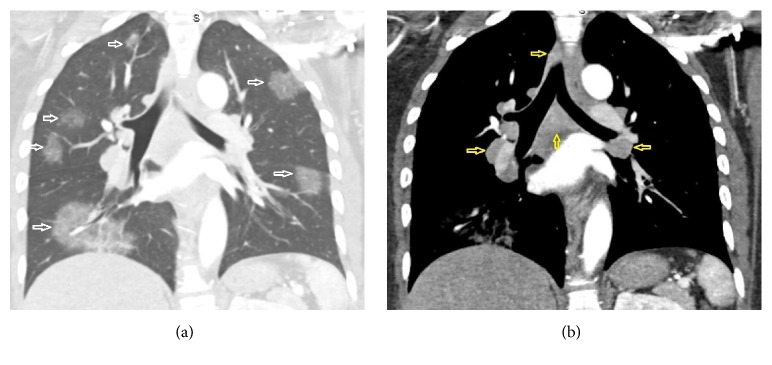
Coronal reformatted image from contrast-enhanced CT scan, with lung window (a), redemonstrating the findings in Figure 2 (white arrows), and with soft tissue window (b) demonstrating lymphadenopathy in the right paratracheal, bilateral hilar, and subcarinal regions (yellow arrows).

An African American woman in her mid-30s presented to the emergency room with neurologic-type symptoms. Initial work-up included a chest X-ray which demonstrated multiple bilateral pulmonary masses and right paratracheal lymphadenopathy (Figure 1). Further evaluation with chest CT (Figures 2 and 3(a)) revealed discrete, sharply demarcated, mass-like ground-glass opacities involving all lobes bilaterally. The CT confirmed the right paratracheal lymphadenopathy and also showed bilateral hilar, para-aortic, subcarinal, and prevascular lymphadenopathy (Figure 3(b)). No interstitial opacities or perilymphatic nodules were identified otherwise. The differential diagnosis based on the imaging findings included lymphoma, vasculitis, and atypical pulmonary infection.

The patient did not report any significant respiratory symptoms. An extensive work-up was performed as the clinical differential included inflammatory, infectious, vasculitic, embolic, and neoplastic etiologies. Rheumatologic, serologic, and infectious work-up were negative. Her angiotensin converting enzyme (ACE) was elevated at 57 (reference range 8–52 U/L).

**Figure 4:**
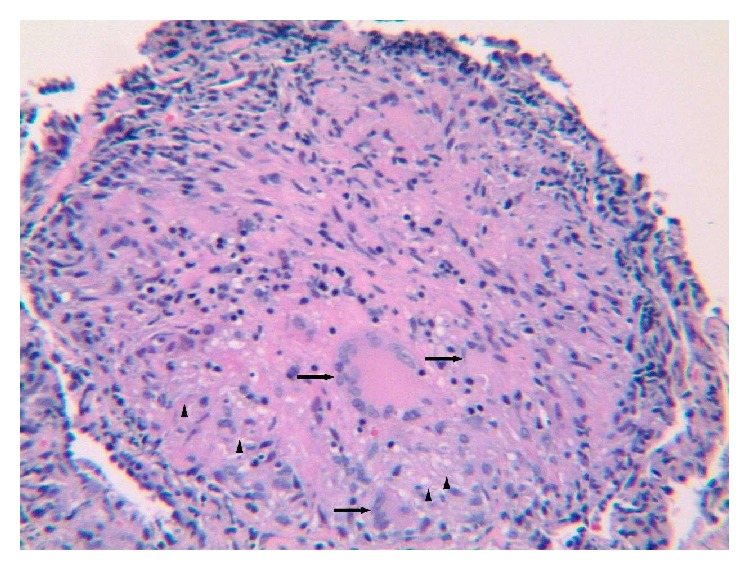
Hematoxylin-eosin stain image from right lower lobe biopsy shows a non-necrotizing granuloma with epithelioid histiocytes (arrowheads) and giant cells (arrows).

She went on to have bronchoscopy. Cultures from bronchoalveolar lavage were negative for bacterial, fungal, mycobacterial, and viral etiologies. There was no evidence of malignancy. Fine-needle aspiration of an enlarged right paratracheal lymph node and biopsy of the right lower lobe (Figure 4) both revealed noncaseating granulomas, consistent with sarcoidosis.

Given the histopathologic findings with the elevated ACE level, the diagnosis was consistent with sarcoidosis and she was started on treatment with corticosteroids.

## 3. Discussion

To the best of our knowledge, this is the first case reporting mass-like ground-glass opacities in a peribronchovascular distribution as a presentation of sarcoidosis. Typical manifestations of thoracic sarcoidosis include bilateral symmetric hilar lymphadenopathy and interstitial lung disease [1]. Atypical manifestations are seen in approximately 25–30% of cases and have been described as mass-like airspace consolidations, miliary opacities, fibrocystic changes, airway involvement, and pleural involvement [4, 5]. Ground-glass opacities are also one of the atypical manifestations in sarcoidosis but have been typically described as diffuse or patchy ill-defined opacities with a background of interstitial nodules. While our case did show some typical findings including right paratracheal and bilateral hilar lymphadenopathy, the discrete mass-like ground-glass opacities have not been described previously. Additionally, parenchymal findings in sarcoidosis are primarily seen within the upper and middle lobes, and in our case all lobes were affected [6]. It is known that up to half of cases of sarcoidosis may be asymptomatic with incidental findings on imaging [1]. While our patient did not have pulmonary-type symptoms, during the patient's hospital course she had elevated troponins which led to a cardiac MRI suggesting preclinical sarcoidosis involvement, and her neurologic symptoms were attributed to neurosarcoidosis. Most patients do not require treatment. Indications for treatment include symptoms, worsening organ damage, or declining pulmonary function. Typically, corticosteroids are commonly used for the initial treatment [7]. A majority of patients will have spontaneous remission, but some may develop a chronic or progressive course, which may ultimately lead to lung transplantation in cases of end-stage disease.

When presented with the thoracic findings of mass-like ground-glass opacities in a peribronchovascular distribution and lymphadenopathy, one may include sarcoidosis in the differential diagnosis if provided with the appropriate clinical picture.

## Data Availability

Data sharing is not applicable to this article as no datasets were generated or analyzed during the current study.
